# Contributions of Electric and Acoustic Hearing to Bimodal Speech and Music Perception

**DOI:** 10.1371/journal.pone.0120279

**Published:** 2015-03-19

**Authors:** Joseph D. Crew, John J. Galvin III, David M. Landsberger, Qian-Jie Fu

**Affiliations:** 1 Department of Biomedical Engineering, University of Southern California, Los Angeles, California, United States of America; 2 Department of Head and Neck Surgery, University of California-Los Angeles, Los Angeles, California, United States of America; 3 Department of Otolaryngology, New York University School of Medicine, New York, New York, United States of America; Sun Yat-sen University, CHINA

## Abstract

Cochlear implant (CI) users have difficulty understanding speech in noisy listening conditions and perceiving music. Aided residual acoustic hearing in the contralateral ear can mitigate these limitations. The present study examined contributions of electric and acoustic hearing to speech understanding in noise and melodic pitch perception. Data was collected with the CI only, the hearing aid (HA) only, and both devices together (CI+HA). Speech reception thresholds (SRTs) were adaptively measured for simple sentences in speech babble. Melodic contour identification (MCI) was measured with and without a masker instrument; the fundamental frequency of the masker was varied to be overlapping or non-overlapping with the target contour. Results showed that the CI contributes primarily to bimodal speech perception and that the HA contributes primarily to bimodal melodic pitch perception. In general, CI+HA performance was slightly improved relative to the better ear alone (CI-only) for SRTs but not for MCI, with some subjects experiencing a decrease in bimodal MCI performance relative to the better ear alone (HA-only). Individual performance was highly variable, and the contribution of either device to bimodal perception was both subject- and task-dependent. The results suggest that individualized mapping of CIs and HAs may further improve bimodal speech and music perception.

## Introduction

Due to relaxing criteria for implantation, increasing numbers of cochlear implant (CI) recipients have some amount of residual acoustic hearing [1]. This acoustic hearing is often aided by a hearing aid (HA), and this "bimodal" listening or "electro-acoustic stimulation" (EAS) has been shown to benefit CI users’ speech perception. CIs provide many patients with good speech understanding in quiet, easy listening conditions. However, CIs do not provide the spectro-temporal fine structure information needed to segregate speech from noise. The coarse spectral resolution also does not support complex pitch perception which is important for musical melody perception and segregation of competing melodies and/or instruments. As a result, many CI listeners have difficulty perceiving and enjoying music [2–4]. Acoustic hearing with HAs provides low-frequency pitch cues that may work in conjunction with a CI to better represent different aspects of sound. Many bimodal listeners report that music and voices sound more natural while listening with a CI and a HA [5–6]. Presumably, the HA restores low-frequency fine-structure cues that provide useful pitch information.

The benefits of combined electric and acoustic hearing for speech perception are well documented [7–15]. In general, combined device performance is better than CI-only performance, but outcomes may be subject- and/or task-dependent. Kong et al. [9] demonstrated that the combined use of HA and CI improved sentence recognition in noise; however, this improvement was not observed at all signal-to-noise ratios (SNRs) or for all subjects. While Brown and Bacon [8] showed improved performance with EAS relative to CI-only, results were mixed for Gifford et al. [12] and Mok et al. [10]. Kong and Braida [16] tested the integration of information across ears and found improved performance in all NH subjects when low-frequency acoustic information was added to vocoded speech; however, only a few CI listeners exhibited such bimodal benefits. Gfeller et al. [17] found no significant difference between hybrid EAS users (acoustic hearing combined with a short CI electrode array in the same ear) and standard electrode length, unilateral CI users when measuring speech performance with adaptive music backgrounds. Zhang et al. [14] and Brown and Bacon [8] demonstrated that otherwise unintelligible low-frequency sounds provide most of the bimodal or EAS benefit, compared with electric hearing only. Taken together, these results suggests that combined electric and acoustic speech performance may benefit even from very limited and crude acoustic information provided by HAs; this limited acoustic information is even more important for music perception.

In contrast to speech recognition, music perception has not been as deeply investigated with bimodal or EAS listeners. Kong et al. [9] tested familiar melody recognition as a function of device type (HA and/or CI) and frequency range. The authors found that different from speech perception, HA performance was better than CI performance for familiar melody recognition. Similar results were found by Dorman et al. [13] in a melody identification task, with HA-only performance better than CI-only and bimodal performance similar to HA-only. Gfeller et al. [18] showed that hybrid EAS subjects outperformed unilateral CI subjects with a standard electrode array in both melody and instrument identification tasks. Gfeller et al. [19] found better pitch discrimination by hybrid EAS subjects, compared with unilateral CI subjects with standard arrays; pitch discrimination was also significantly correlated with familiar melody recognition. Gfeller et al. [20] measured melody identification using real-world music excerpts and found a benefit when acoustic hearing was added to a CI. Kong et al. [21] used multi-dimensional scaling (MDS) to examine timbre perception in bimodal and bilateral CI users, and found no advantage to combined use of CI+HA or two CIs, compared with the better ear alone; the authors remarked that the dominant cue for timbre was the spectral envelope which was sufficiently transmitted by a single CI.

El Fata et al. [22] divided bimodal subjects into two groups according to the audiogram of the acoustic ear. Using a melody identification task with and without lyrics, the authors found improved performance with CI+HA (relative to the CI alone) only for the group with better acoustic hearing. Interestingly, subjects in the group with poorer acoustic hearing preferred to listen to music with only the CI while the group with better acoustic hearing preferred to listen to music with both the CI and the HA. Looi et al. [23–24] found that CI users rated music as more pleasant sounding than HA-only subjects, even though HA users outperformed CI users on pitch and melody tasks. Looi and Radford [25] found no significant difference between pitch ranking scores between bimodal and unilateral CI users, however, the lowest tested interval was a quarter octave, 3 semitones. These studies demonstrate a widely ranging contribution of acoustic hearing to bimodal and EAS users’ music perception that seems to depend on several factors including (but not limited to) the amount of residual acoustic hearing, the listening task, CI-only performance, etc. However, it is unclear how acoustic cues contribute to CI music perception. Presumably, the added acoustic information should improve melodic pitch perception and/or segregation of competing melodies.

In this study, speech and music perception were measured in bimodal subjects listening with the CI only, the HA only, or both devices (CI+HA). Sentence recognition was measured in competing multi-talker babble. Melodic pitch perception was measured using a melodic contour identification task (MCI) [26–27]. The MCI task was used to provide some quantification of bimodal subjects’ functional pitch perception across device conditions. The fundamental frequency (F0) range of the target contours was constrained to be audible with the HA and with the CI. MCI performance was also measured with a competing masker; the masker F0 range either overlapped with that of the target contour (i.e., audible with the HA and the CI), or was remote from the target (i.e., audible with only the CI). We hypothesized that combined use of CI+HA would provide better speech and music performance than with either device alone. We also hypothesized that for the MCI task, segregation of the masker and target would be easier when the masker F0 range did not overlap with the target F0 range.

## Methods

### Subjects

Eight bimodal subjects, those with a CI in one ear and a HA in the opposite ear, participated in the study. All but one of the subjects had participated in a previous speech related bimodal study [15], and the final subject was recruited among the general population. Table 1 shows subject demographic information. The only inclusion criteria were that the subjects used both devices on a daily basis and had more than one year of bimodal listening experience. No subject was excluded on the basis of speech scores, acoustic hearing audiogram, musical experience, etc. No subjects had any formal music training; however, subjects S1, S2, and S3 had previously participated in an MCI training study [28]. In that study, only the CI was trained and tested; in the present study, the testing of HA and CI+HA was novel. All subjects were post-lingually deafened except for S5 who was peri-lingually deafened. Thus, the subject pool represented a broad range of bimodal users: with a range speech scores, with and without musical training, different device type, etc.

**Table 1:** Subject demographic information.

Subject	Age	Onset of Hearing Loss (Years)	CI Experience (Years)	CI	HA	Etiology of Hearing Loss
S1	79	14	13	Advanced Bionics	Phonak	Sudden Sensorineural
S2	79	35	12	Cochlear	Siemens	Sensorineural Progressive
S3	75	31	3	Cochlear	Resound	Noise Exposure
S4	43	22	2	Cochlear	Phonak	Sensorineural Genetic
S5	47	47	14	Cochlear	Phonak	German Measles
S6	70	8	1	Advanced Bionics	Oticon	Meniere's Disease
S7	59	10	8	Advanced Bionics	Oticon	Ototoxicity
S8	65	35	8	Advanced Bionics	Widex	Cochlear Otosclerosis
S9	79	15	1	Cochlear	Oticon	Familial

### Ethics statement

This study and all experimental procedures were approved by the Institutional Review Board at St. Vincent's Hospital in Los Angeles, CA, USA at the time of testing. Each subject provided written consent for participation in the study. Subjects were informed of the risks and potential rewards before testing began. Subjects were compensated for their time each test day.

### Audiometric thresholds

**Fig 1:**
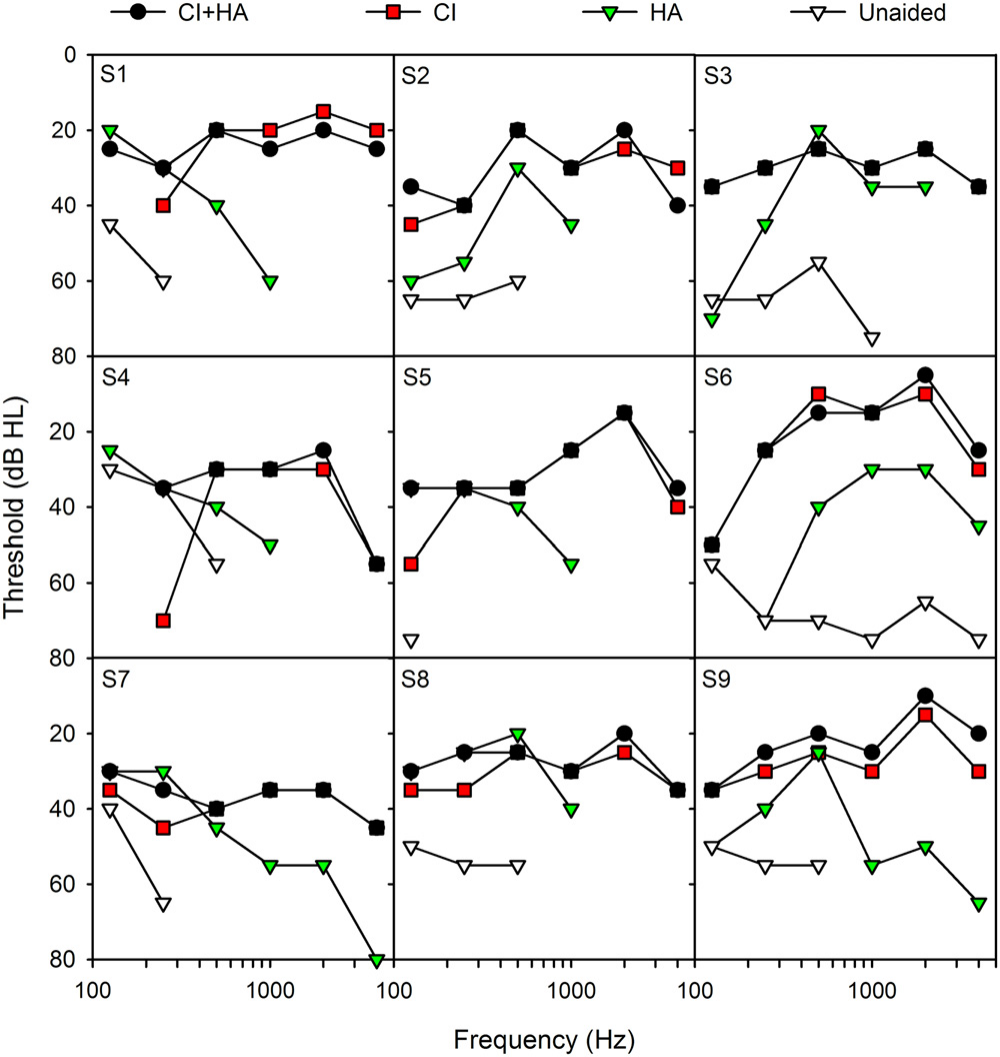
Audiometric thresholds for each subject for different hearing devices. CI+HA (black circles), CI (red boxes), HA (green triangles), and unaided (white triangles) thresholds are shown for each subject. Thresholds greater than 80 dB HL are not shown.

Fig. 1 shows thresholds across different listening conditions for each subject. Audiometric thresholds using warble tones were collected in sound field with the CI+HA, CI only, HA only, and unaided for each subject. For the CI, HA, and CI+HA conditions, thresholds were measured using subjects’ clinical devices and settings. Subjects sat in a sound-treated booth (IAC) approximately 1 m away from and facing towards a single loudspeaker. CI+HA thresholds were collected first. Next, CI-only thresholds were collected; the HA was removed and that ear was plugged. Next, HA-only thresholds were collected; the CI speech processor was removed and that ear was plugged. Finally, unaided thresholds were collected by removing both devices, but plugging neither ear.

### General procedure

All testing was performed with the CI only, the HA only, and CI+HA. The test order for device conditions was randomized within and across subjects. Subjects used their clinical CI and HA device and settings. To ensure that loudness was perceived as similar across devices, subjects listened to a few melodic contours while using the CI and the HA. If a subject indicated that the HA was lower in volume relative to the CI, the subject was instructed to increase the HA volume, and this setting was used for all testing. All subjects (except for S1) indicated that the loudness was approximately equal across devices and made no adjustment to either device; subject S1 slightly increased the volume of the HA to match the loudness of the CI.

### Speech stimuli and testing

Speech understanding in noise was tested using sentences from the Hearing in Noise Test (HINT) [29] presented in multi-talker speech babble. Speech reception thresholds (SRTs) were adaptively measured. The SRT was defined as the signal-to-noise ratio (SNR) that yields 50% words correctly identified in sentences (Rule 3 from Chan et al. [30]). The speech level was fixed at 65 dBA, and the level of the background noise was varied according to subject response. The initial SNR was 20 dB. If the subject correctly identified 50% or more of the words in the test sentence, the background noise was increased by 2 dB. If the subject identified less than 50% of the words in the test sentence, the background noise was reduced by 2 dB. A minimum of three runs were collected for each hearing mode condition.

### Music stimuli and testing

Melodic pitch perception was tested using a melodic contour identification (MCI) task [26–27]. In the MCI task, a subject is presented with one of nine possible target contours (“rising,” “falling,” “flat,” “rising-flat,” “falling-flat,” “rising-falling,” “falling-rising,” “flat-rising,” and “flat-falling”) and is asked to identify which contour was presented. Each contour consisted of 5 notes, 300 ms in duration with a 300 ms silent interval between notes. The semitone spacing between consecutive notes in the contour was varied from 1 to 3 semitones, allowing for some measure of pitch resolution. The lowest note for each target contour was 220 Hz (A3) with a highest possible note of 440 Hz (A4). As such, the F0 range of the target contours was audible with both the HA and the CI. Each note for the target contour was played by a MIDI synthesized piano sample. The piano was chosen because it produced the lowest scores in previous MCI studies [31] as it had the most complex spectro-temporal envelope.

**Fig 2:**
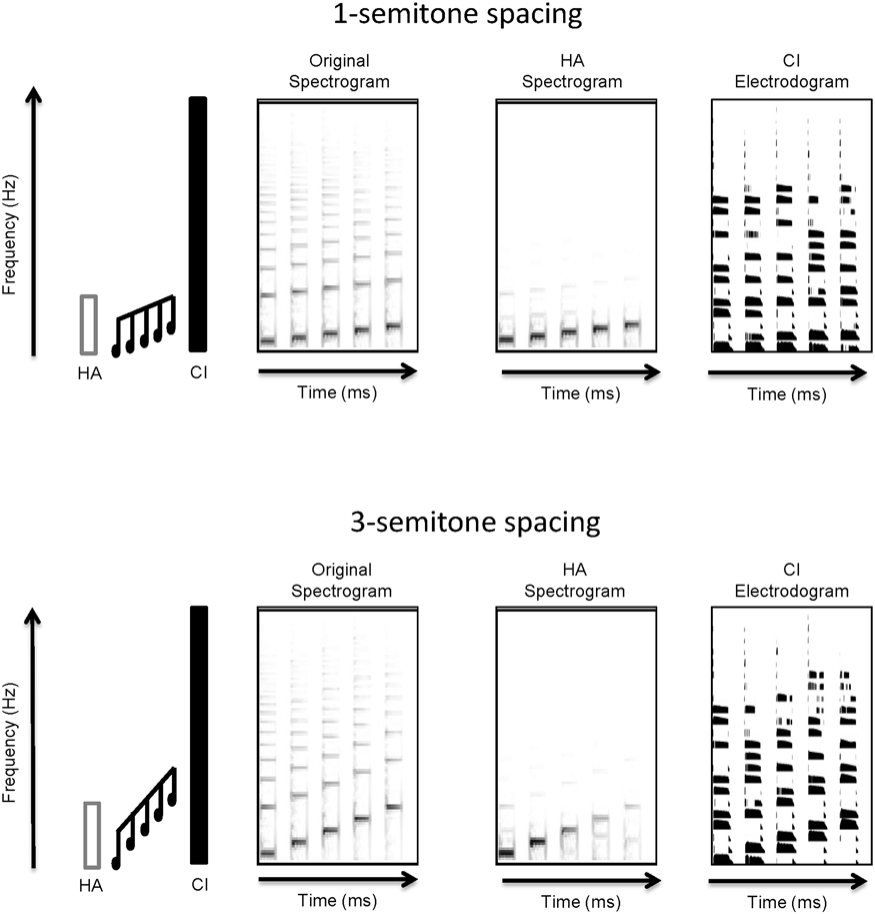
Spectrograms and electrodograms for the No Masker condition for 1- and 3-semitone spacings. The far left panel shows a schematic representation of HA and CI frequency ranges. The target contour is shown in black. The middle two panels show a spectral representation of the original stimuli (left) and simulated HA output (right). A steeply sloping hearing loss was simulated using AngelSim and is intended for illustrative purposes only. The far right panel shows an idealized electrodogram representing the electrical stimulation patterns for a CI. Electrodograms were simulated using default stimulation parameters for the Cochlear Freedom and Nucleus-24 devices: 900 Hz/channel stimulation rate, 8 maxima, frequency allocation Table 6, etc.

Fig. 2 shows the “rising” contour with 1-semitone (top row) or 3-semitone (bottom row) spacing. The far left side of Fig. 2 illustrates the different contours within the HA and CI frequency ranges. The original spectrogram of the contours is shown just to the right; differences in the extent of F0 range can be seen between the 1- and 3-semitone spacing conditions. Next right is a spectrogram of the contours processed by a hearing loss simulation (AngelSim from www.tigerspeech.com). A steeply sloping hearing loss was simulated (0 db HL at 125 Hz, 20 dB HL at 250 Hz, 60 db HL at 500 Hz, 60 dB HL at 1000 Hz, 100 dB HL at 2000 Hz, 120 dB HL at 4000 Hz, and 120 dB HL at 8000 Hz) for illustrative purposes only, and was not intended to represent any subject’s audiogram. Differences in high frequency harmonic information can be easily seen between the original and HA spectrograms. The far right of Fig. 2 shows electrodograms that represent the electrical stimulation patterns given the default stimulation parameters for the Cochlear Freedom and Nucleus 24 devices which employs an 8 of 22 channel selection strategy. [The electrograms for subjects S1, S6, S7, and S8 would be slightly different as they use an Advanced Bionics device (16 channels; no channel selection).] The y-axis represents electrodes from the apex (bottom) to the base (top). Differences in the stimulation pattern across notes can be seen with the 3-semitone spacing (bottom); with the 1-semitone spacing (top), the changes in the stimulation pattern are more subtle.

MCI was tested with and without a competing instrument masker. The masker was played by MIDI synthesized clarinet sample similar to Galvin et al. [31]. Different from the piano, the spectral and temporal envelopes were less complex for the clarinet, allowing for potential timbre differences between the masker and target, as in Zhu et al. [32]. The masker contour was always “flat” (i.e., the same five notes), and was presented simultaneously with the target contour (i.e. the same onset and offset), as in Galvin et al. [33]. The masker frequency range was varied to be overlapping (A3 masker, 220 Hz) or non-overlapping (A6 masker, 1760 Hz) with the target. The A3 masker was audible with the HA and the CI, and the A6 masker was audible only with the CI for most subjects.

**Fig 3:**
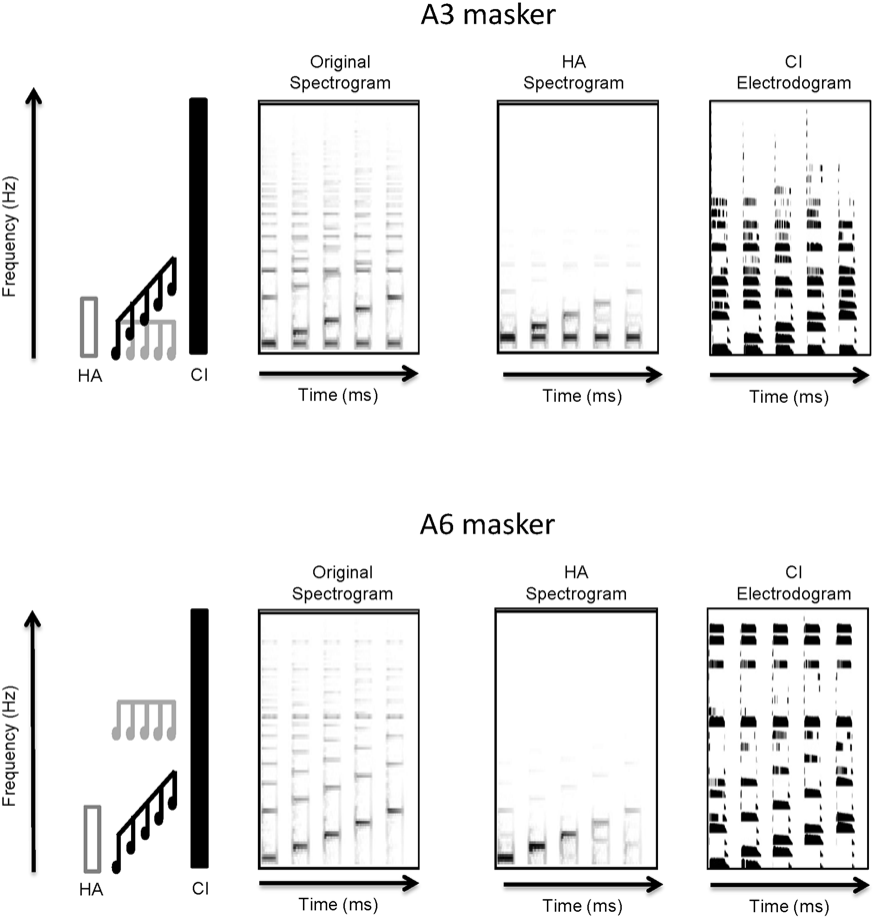
Spectrograms and electrodograms for the A3 and A6 Masker conditions. The top half of the figure shows (from left to right) a schematic representation of the test condition in relation to the frequency ranges of the HA and the CI, a spectrogram of the original stimuli, a spectrogram of the simulated HA output, and an idealized electrodogram for the A3 Masker condition; the bottom half shows the same information for the A6 Masker condition. Figure details are similar to the details of Fig. 2. The target instrument notes are shown in black and the masking instrument notes are shown in gray.

Fig. 3 shows the “rising” contour with 3-semitone spacing with the overlapping A3 masker (top row) and the non-overlapping A6 masker (bottom row). Fig. 3 is similar in presentation to Fig. 2. The far left side of Fig. 3 illustrates the masker and target contours in relation to the HA and CI frequency ranges; the A6 masker is shown to be in the CI range only. The original spectrogram is shown just to the right. The overlapping masker can be seen at the bottom of the A3 original spectrogram, and the non-overlapping masker can be seen above the changes in F0 in the A6 original spectrogram. The HA spectrograms show that only the A3 overlapping masker is represented in the simulated audible acoustic hearing range. The electrodograms at far right show the A3 and A6 maskers simultaneously presented with target contours. Note also the differences in the stimulation patterns with the maskers in Fig. 3 to the target alone (bottom panel of Fig. 2).

All MCI stimuli were presented acoustically at 65 dBA in a sound-treated booth. During each test block, a contour was randomly selected (without replacement) from among the 54 stimuli (9 contours * 3 semitone spacings * 2 repeats) and presented to the subject, who responded by clicking on one of the nine response boxes shown onscreen. Subjects were allowed to repeat each stimulus up to three times. Some subjects (S1, S2, S3) were familiar with the MCI task from previous experiments. For the remaining subjects, the response screen and test procedures were carefully explained to the subjects. No preview or trial-by-trial feedback was provided. A minimum of three test blocks were tested for each hearing mode condition and masker condition; if the variability in performance was greater than 20%, a fourth run was performed. For each condition and subject, performance was averaged across all runs.

## Results

**Fig 4:**
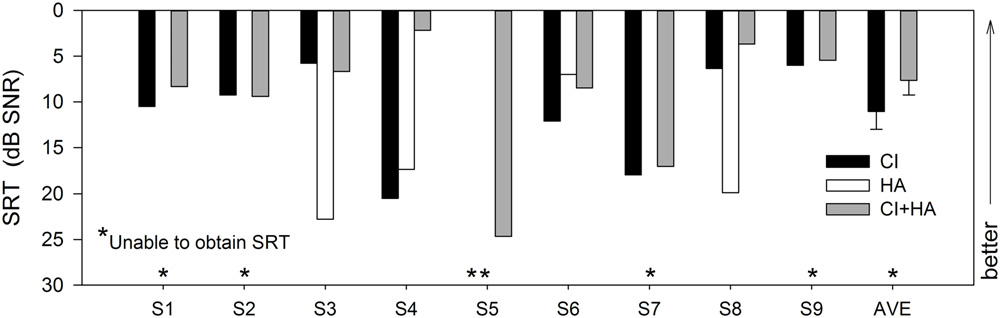
Speech-in-noise results for individual subjects across hearing devices. CI-only SRTs are shown by the black bars, HA-only SRTs are shown by the white bars, and CI+HA SRTs are shown by the gray bars. Mean performance is shown at the far right; error bars indicate standard error. Asterisks indicate that SRTs could not be measured for that condition. Bars closer to the top of the graph indicate better performance.

Fig. 4 shows individual subjects’ SRTs across the different listening conditions; mean performance is shown at the far right. SRTs with the HA-only could not be obtained for subjects S1, S2, S5, S7, and S9, as performance was <50% correct for SNRs >30 dB; similarly, the CI-only SRT could not be obtained for subject S5. While the missing SRTs make the statistical analyses difficult, they do in fact represent a particular level of speech understanding. Failure to obtain an SRT for SNRs > 30 dB suggests that the subject most likely could not correctly identify 50% of the words in quiet. In general, speech performance was much poorer with the HA than with the CI. For most subjects, CI+HA speech performance was comparable to CI-only performance. For subject S4, CI+HA performance was much better than with either device alone. A one-way repeated-measures analysis of variance (RM ANOVA) showed no significant of device type (CI-only vs. CI+HA) [*F*(1,14) = 1.8, *p* = 0.201]; however, observed power was low (0.125). HA-only SRTs were not included in the analysis as we were unable to obtain SRTs for a number of subjects; S5 was also excluded from the analysis as there was no CI-only SRT for this subject.

**Fig 5:**
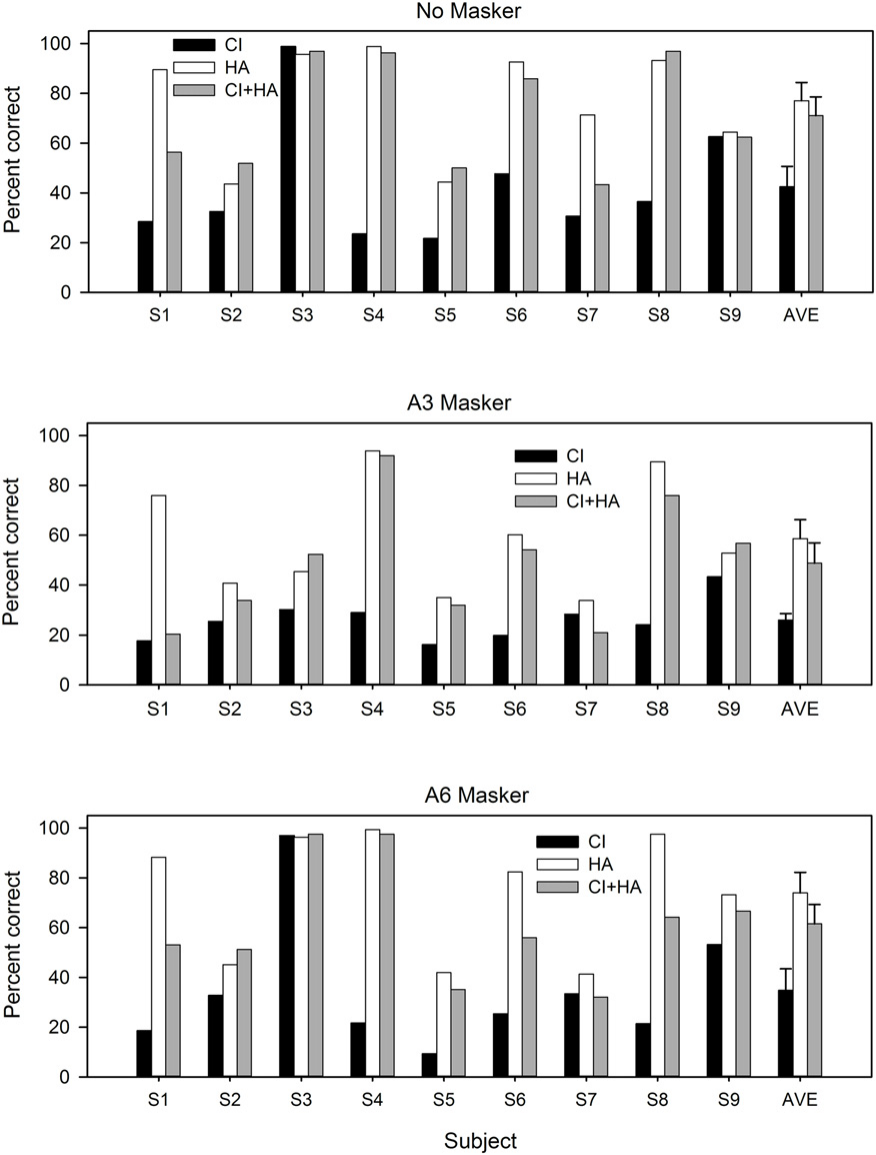
MCI performance for individual subjects across hearing devices and masker condition. CI-only performance is shown by the black bars, HA-only performance is shown by the white bars, and CI+HA performance is shown by the gray bars. Mean performance is shown at the far right within each masker condition; error bars indicate standard error. MCI with No Masker is shown in the top panel, MCI with the overlapping, A3 Masker is shown in the middle panel, and MCI with the non-overlapping, A6 Masker is shown in the bottom panel.

Fig. 5 shows individual subjects’ MCI performance for the different listening and masker conditions; mean performance is shown at right. Data in Fig. 5 were averaged across the semitone spacing conditions. For all masker conditions, MCI performance was generally best with HA-only and worst with CI-only. Only subjects S3 and S9 performed better than 50% correct with the CI-only for any masker condition; chance performance was 11.1%. CI+HA performance relative to best ear performance was variable across subjects. For example, subject S1 seemed to experience perceptual interference (CI+HA < HA) when listening with both devices in some masker conditions, and subject S4 seemed to focus on the better ear (CI+HA ≈ HA). Some subjects (S2 and S5) seemed to experience additive integration in bimodal listening (CI+HA > HA); interestingly these were the poorest performing subjects overall. A three-way RM ANOVA showed significant main effects for hearing mode [*F*(2,16) = 12.7, *p* < 0.001], masker condition [*F*(2,16) = 8.5, *p* = 0.003], and semitone spacing [*F*(2,16) = 25.1, *p* < 0.001]. The observed power was greater than 0.9 for all main effects. A significant interaction was observed only between masker condition and semitone spacing [*F*(4,32) = 3.3, *p* = 0.022] with an observed power of 0.781. Post-hoc paired t-tests revealed significant differences after Bonferroni corrections (α_crit_ = 0.017) for the following: CI vs. HA (*p* = 0.0042), CI vs. CI+HA (*p* = 0.0087), No Masker vs. A3 Masker (*p* = 0.0065), 1 Semitone vs. 2 Semitones (*p* = 0.0055), and 1 Semitone vs. 3 Semitones (*p* < 0.001).

**Fig 6:**
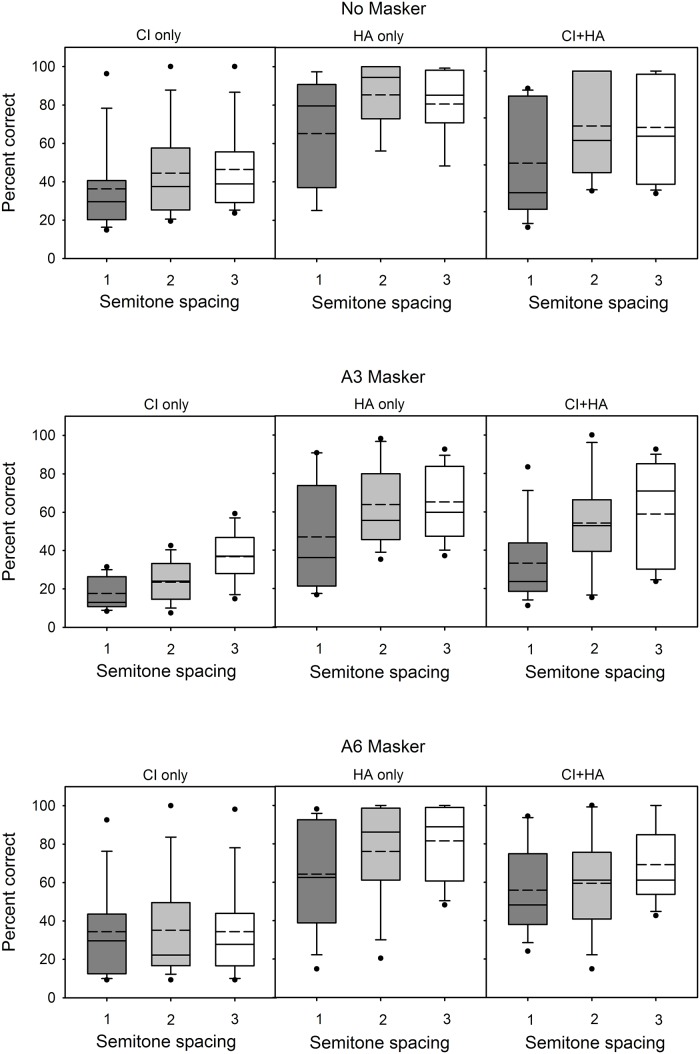
Boxplots of MCI performance as a function of semitone spacing, for the different listening and masker conditions. The columns indicate hearing device (CI, HA, and CI+HA) and the rows indicate masker condition (No Masker, A3 Masker, A6 Masker). The edges of the boxes represent the 25th and 75th percentiles, the solid line represents the median, the dashed line represents the mean, the error bars indicate the 10th and 90th percentiles, and the points outside of the error bars indicate outliers.

Fig. 6 shows the effect of semitone spacing on MCI performance for different hearing mode and masker conditions. CI-only performance was generally poor and less variable (except for outlier S3) compared with HA-only and CI+HA performance; this was most likely due to floor effects. Performance generally improved as the semitone spacing increased. Performance with the HA alone was much better but also more variable across subjects, relative to CI-only performance. HA-only performance generally improved with increasing semitone spacing. CI+HA performance was generally poorer with 1-semitone spacing, and better with the 2- and 3-semitone spacing conditions (though there was little difference between the 2- and 3-semitone spacings). With 1-semitone spacing, the mean HA performance for the No Masker condition was 65.2% correct, while the mean HA performance for the A3 overlapping masker condition was 46.9% correct. This suggests that subjects had difficulty segregating the overlapping contours even when fine structure cues were available in the HA. With 1-semitone spacing, mean CI+HA performance for the No Masker and the A6 Masker conditions was 60.7% and 55.8% correct, respectively. This suggests that listeners were able to selectively attend to the target when the masker was spatially remote. A similar pattern of results was observed with the HA-only for the No Masker and A6 masker conditions.

Correlational analyses were performed among the different hearing conditions; data was collapsed across the No Masker, A3 masker, and A6 masker conditions. There was no significant correlation between CI-only and HA-only performance (r^2^ = 0.085, *p* = 0.140). There was a significant correlation between CI-only and CI+HA performance (r^2^ = 0.247, *p* = 0.008); however, the correlation was not significant when outlier S3 was removed from the analysis (r^2^ = 0.074, *p* = 0.190). There was a significant correlation between HA-only and CI+HA performance (r^2^ = 0.615, *p* < 0.001).

**Fig 7:**
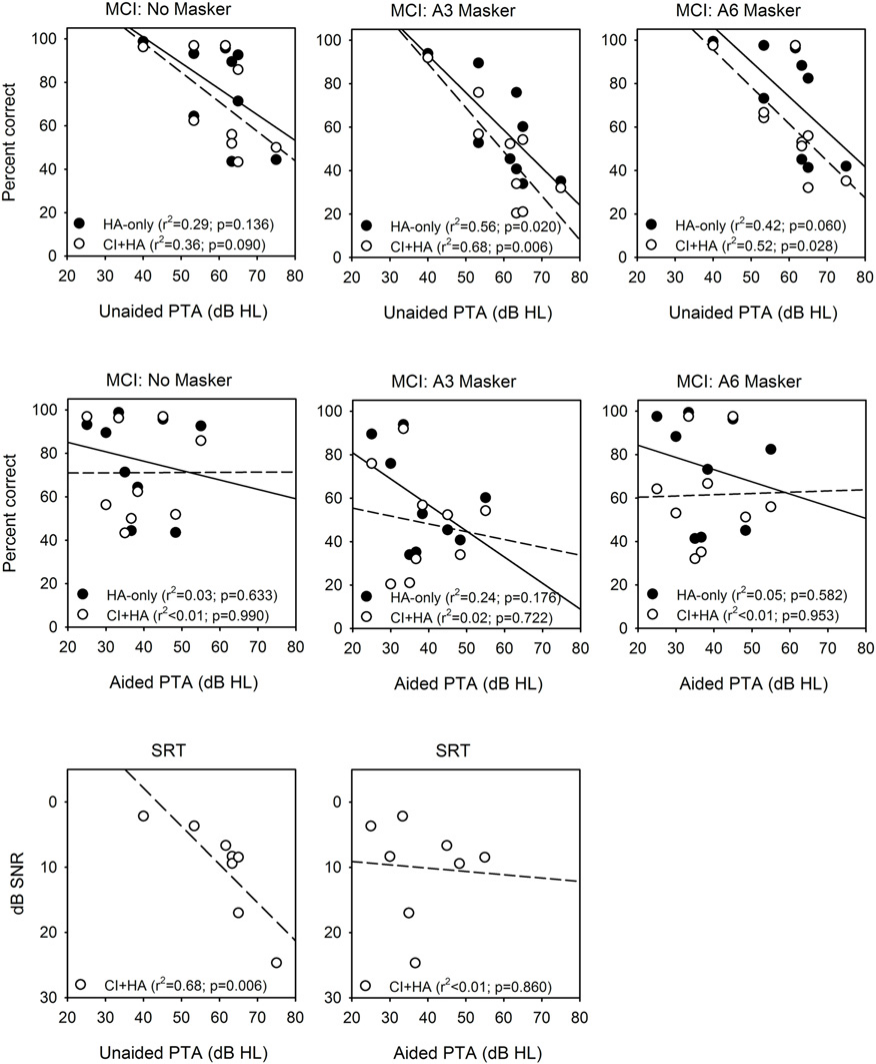
Scatter plots of music and speech performance versus unaided and aided thresholds in the non-implanted ear. The top row shows MCI performance for the No Masker (left), A3 Masker (middle), and A6 Masker conditions (right), as a function of unaided PTAs at 125 Hz, 250 Hz, and 500 Hz. The solid circles show data for the HA-only condition; the solid line shows the linear regression (r^2^ and p-values are shown in the legend in each panel). The open circles show data for the CI+HA condition; the dashed line shows the linear regression. The middle row shows similar plots, but as a function of aided PTAs at 125 Hz, 250 Hz, and 500 Hz. The bottom row shows SRTs as a function of unaided PTAs (left) or aided PTAs (middle). Only CI+HA SRT data is shown.

Fig. 7 shows music and speech perception as a function of unaided and aided thresholds in the non-implanted ear. Pure-tone average thresholds (PTAs) were calculated for each subject at 125 Hz, 250 Hz, and 500 Hz; note that warble tones were actually used to measure sound field thresholds. Linear regressions were performed for to the HA-only and the CI+HA data. Results showed that unaided PTAs were moderately correlated with speech and music performance. There were significant correlations between unaided PTAs and MCI performance for a few conditions [HA-only, A3 masker (*p* = 0.020); CI+HA, A3 masker (*p* = 0.006); CI+HA, A6 masker (*p* = 0.028)]. Unaided PTAs were also significantly correlated with CI+HA SRT scores (*p* = 0.006). However, there were no significant correlations between aided PTAs and speech or music performance.

## Discussion

The present data show that the contributions of acoustic and electric hearing to bimodal performance were both subject- and task-dependent. Similar to Kong et al. [9], we found that the CI and the HA provide differing types and amounts of information for bimodal listeners, with speech information mostly provided by the CI and low-frequency pitch information provided by the HA. There was evidence to support the hypothesis that combined use of CI+HA provided better performance than with either device alone for speech but not for music. Indeed, mean MCI performance for all masker conditions was poorer (though not significantly poorer) with CI+HA than with the better ear (HA). Similarly, the advantage with CI+HA versus CI-only was not significant for SRTs in babble. Thus, the hypothesis that adding a second (presumably poorer-performing) device would improve performance was not supported; mean HA performance was greater than mean CI+HA performance across masker conditions. Likewise, our hypothesis that combined use of CI+HA would improve segregation of competing melodic contours was not supported. There was some evidence to support our hypothesis that separating the F0 range of the masker and target contours (effectively isolating the masker on the CI side) would improve MCI performance with a competing instrument (e.g., data from subjects S1, S2, and S3). However, performance with the A6 masker was not significantly better than with the A3 masker (*p* = 0.060). Also, there was no significant difference between the A6 masker and No Masker conditions, suggesting that listeners were able to selectively attend to the target presented on the HA side even when a masker was presented only to the CI side.

As seen in other studies [9–10,12,16,19, 22], there was considerable across-subject and even within-subject variability in the present data. A subject might perform well in one task, but poorly in another. For example, subject S3 performed very well in the No Masker and A6 Masker conditions but poorly in the A3 Masker condition. Likewise, subject S2 was a middling performer in the speech task but one of the poorer performers in the music tasks. Interestingly, the across-subject variability was increased in the MCI tasks for the HA-only and CI+HA conditions, relative to that with the CI-only (see Fig. 6). Performance tended to be uniformly poor with the CI-only, with a much wider range in performance with the HA-only or the CI+HA. CI+HA performance was greater than CI-only performance in nearly all cases.

### Bimodal speech perception

In this study, mean SRTs with the CI+HA were slightly (but not significantly) better than with the CI-only (3.4 dB difference on average, *p* > .05). Dorman et al. [13] found significantly better performance with the CI+HA relative to CI-only for CNC words and AzBio sentences. Brown and Bacon [8] also found significantly better sentence recognition scores with CI+HA relative to CI-only. Mok et al. [10] found significantly better performance with CI+HA relative to CI-only for CNC phonemes and CUNY sentences at +10dB SNR, but not for CNC words and CUNY sentences at +5dB SNR. Mok et al. [10] also found significantly better SRTs with CI+HA versus CI-only; note that these authors measured closed-set spondee identification in noise whereas open-set sentence recognition was used in the present study. While the present data does not show a significant advantage with the CI+HA relative to CI-only, the statistical power was very low.

For most subjects, the HA provided very little speech information. It is not surprising that HA-only performance was poor; if speech understanding was sufficiently good with the HA alone, subjects would not have qualified for cochlear implantation. It was not possible to obtain SRTs in some subjects (S1, S2, S5, S7, S9), even at very high SNRs. Interestingly, some subjects (S4, S6) demonstrated substantial speech understanding in noise with the HA alone; in these subjects, performance with the HA alone was better than with the CI alone. The variability in HA-only performance may reflect differences in auditory processing other than audibility (e.g., spectral resolution, temporal processing, etc.). Zhang et al. [34] recently found that the spectral resolution within the residual acoustic hearing, rather than the range of audibility, predicted the benefit of combined use of CI+HA. Aided and unaided thresholds (Fig. 1) suggest that HA signal processing may have differed across subjects. For example, S6 had the most high frequency hearing in the unaided condition and had the best HA-only SRTs; likewise, S5 had the worst unaided audiogram and was generally the poorest performer.

### Bimodal music perception

While bimodal speech performance seemed to be largely driven by the CI, bimodal music performance seemed to be largely driven by the HA. Mean MCI performance for the No Masker condition was 77.0% correct with the HA-only and 42.4% correct with the CI-only. Excluding “star” subject S3, CI-only scores ranged from 21 to 62% correct while HA-only scores ranged from 43 to nearly 100% correct in the No Masker condition. CI+HA scores tended to be very similar to HA-only scores for most subjects; some subjects (S1, S7) showed considerably poorer performance with CI+HA relative to HA-only. From Fig. 6, the resolution of the HA is better than 1-semitone for most subjects; excluding S3, the resolution of the CI is worse than 3-semitones. For all masker conditions, mean MCI performance with CI+HA was slightly poorer than with the HA alone, and poorer still with the CI alone. In contrast, Kong et al. [9] found that familiar melody identification was on average better with CI+HA than with HA-only; interestingly some subjects performed better with the CI-only than with the HA-only. Differences in the listening tasks (MCI vs. familiar melody recognition) may explain difference in outcomes between studies.

The effect of the maskers was somewhat variable across subjects. On average, performance was worse with the A3 Masker than with the A6 Masker. Mean performance dropped by 6.8% from No Masker to A6 Masker conditions; this deficit was not significant after family-wise type I error correction (paired t-test with Bonferroni correction: *p* = 0.033, α_crit_ = 0.017). Mean performance significantly dropped by 19.1% from No Masker to A3 Masker (paired t-test with Bonferroni correction: *p* = 0.007). Some subjects experienced a large drop in performance for particular masker conditions. For example, subject S3 scored nearly perfectly in all hearing modes for the No Masker and A6 Masker conditions, but experienced a very large drop in performance in the A3 Masker condition. Other subjects had similar performance in the A6 Masker and A3 Masker conditions (e.g., S4, S5) for all hearing configurations. And for many subjects, the effect of a competing instrument depended on which device was tested. For subject S1, there was a large drop in CI+HA performance between the No Masker and A3 Masker conditions, but only a moderate drop in HA-only performance from the No Masker to the A3 Masker condition. Taken together, these results suggest that the devices contributed differently to individual subject performance, depending on the particular listening task.

### Audibility and perception

As shown in Fig. 7, unaided PTAs were sometimes predictive of speech and music performance while aided PTAs were not. This suggests that the HA signal processing, as applied to these particular subjects, may not have been optimal or consistent across subjects. For example, the HA gain applied to low frequencies was different across subjects despite similar unaided thresholds. Subjects S2, S3, and S6 all had poor unaided thresholds (>60 dB HL) but received no amplification at 125 Hz. In contrast, subjects S1, S5, and S8 received 15 dB or more gain at 125 Hz. Large amounts of gain were sometimes applied at higher audiometric frequencies, most likely to improve speech perception with the HA. However, such high levels of gain may introduce distortion, which may reduce music perception and enjoyment. The good predictive power of the unaided PTAs versus the poor predictive power of the aided PTAs suggests some disconnect between audibility and intelligibility in HA fitting and/or signal processing. To the extent that the unaided thresholds represent peripheral abilities or limits, HA processing must extend these limits without sacrificing performance. This processing might be very different for speech and music, especially in the case of bimodal listening.

The above results demonstrate a number of implications for mapping bimodal listeners. Audibility in the acoustic ear seems largely responsible for melodic pitch perception. S5 was one of the worst performing subjects and had the least amount of residual acoustic hearing. S4 had the best unaided threshold at 125 and 250 Hz and likewise was the best performing subject with the HA alone across all masker conditions. This is consistent with El Fata et al. [22] and Zhang et al. [34], who demonstrated that the audibility and the resolution of the acoustic ear is clearly linked to bimodal performance.

It seems intuitive that audibility in the acoustic ear would contribute strongly to music perception performance. However, there was no clear pattern in the present results that linked acoustic thresholds, aided or unaided, to MCI performance. Audibility alone as measured by pure-tone thresholds may not explain HA-aided performance. The HA prescription may differ greatly among bimodal users, and this may greatly affect music perception with acoustic hearing. The aided and unaided thresholds in Fig. 1 reveal different gain settings across frequencies for different subjects. For example, S1 exhibits a half-gain rule across low frequencies such that the F0s used in this study would have been highly audible with the HA. S3 exhibits a very different HA mapping such that the F0s would have been much less audible with the HA. It is unlikely that a greater number of subjects would improve the correlation between aided thresholds and MCI performance. There needs to be consistent control of HA mapping in order to examine the contribution of aided low frequency hearing to music perception as this mapping has been shown to have an effect on bimodal speech perception [35]. While the present number of subjects is small (n = 9), it is unlikely that a greater number of subjects would change the fundamental relationships between acoustic hearing in speech and acoustic hearing in music for bimodal users. Future studies with a greater number of subjects should consider both unaided thresholds and the amount and type of amplification scheme to better reveal the contributions from the acoustic ear.

### Real-world music listening

The present data, obtained under specific and very constrained speech and music conditions, showed no statistically significant advantage for bimodal listening over the better device alone. Indeed, mean MCI performance was poorer with CI+HA than with HA-only, and many subjects performed equally well with the CI+HA as with the HA-only. However, a number of subjects experienced a considerable performance drop when listening with both devices. In all three masker conditions, HA-only scores were well above the CI+HA scores for subject S1. Subjects S6 and S8 also experienced a drop in performance between HA-only and CI+HA for the A6 Masker condition. Galvin and Fu [36] found that high-pass filtering MCI stimuli improved performance. The authors suggested that the improvement may have been due to reducing the frequency-to-place mismatch for low-frequency F0s, or to de-emphasizing apical pitch cues that may have been less salient. Zhang et al. [14] found that reducing the frequency overlap between the acoustic ear and the CI ear did not improve speech perception for bimodal listening. However, some bimodal listeners (e.g., S1 from the present study) might benefit from reducing low-frequency stimulation from the CI rather than reducing the high-frequency information from the HA. Such an optimization might reduce the perceptual interference between devices for pitch perception. Thus, coordinating the input frequency ranges between devices may improve bimodal listening.

Although the present study found that mean MCI performance was slightly worse with CI+HA than with the HA-only, most subjects indicated that they regularly wear both devices especially when listening to music. With the CI-only, subjects reported that the sound quality was “artificial” and “alien.” Adding the HA made the sound quality more “natural.” The present results must also be considered in terms of the limited materials and measures. Clearly, music contains many more complex components than the simple melodic contours tested in the present study. Music often contains complex timbres, melodies, harmonies, and rhythmic patterns as well as large dynamic and frequency ranges. Therefore, the results of the present study should be interpreted cautiously with regard to more real-world music listening. The frequency range of much music may be inaudible with an HA (e.g., cymbals, higher frequency notes, lyrics), and will be largely represented by the CI. CIs have also been shown represent instrument timbre similarly to NH [37]. Kong et al. [21] found that both the HA and CI represent the attack of an instrument well, but HAs do not represent the spectral envelope of the instrument as well as a CI. El Fata et al. [22] and Gfeller et al. [38] showed that CIs better represent lyrics, which are a very important component of popular music. Thus, combined device use remains likely to produce the best outcomes for music perception and enjoyment.

## Conclusion

In the present study, speech perception in noise and melodic pitch perception were measured with the CI alone, the HA alone, and CI+HA. Results showed

- Mean SRTs were best with CI+HA. However, there was no significant difference in SRTs between the CI+HA and CI-only conditions, suggesting that the HA contributed little to bimodal speech perception.
- Mean MCI performance was best with HA-only, with the CI+HA producing slightly (but not significantly) poorer performance. This suggests that the CI contributed very little to bimodal melodic pitch perception, and in some cases, negatively impacted bimodal MCI performance.
- The presence of a competing instrument reduced MCI performance, especially when the masker and target F0s were overlapping.
- The results demonstrate that different types of information are best represented by the different devices. Individual bimodal listeners may attend to these cues differently, depending on the listening demands.
